# A new marker predicting gestational diabetes mellitus: First trimester neutrophil/lymphocyte ratio

**DOI:** 10.1097/MD.0000000000030511

**Published:** 2022-09-09

**Authors:** Murat Sahin, Ayten Oguz, Dilek Tüzün, Okay Işiktaş, Songül Işiktaş, Cansu Ülgen, Hatice Şahin, Kamile Gul

**Affiliations:** a Faculty of Medicine, Department of Endocrinology and Metabolism, Kahramanmaras Sutcu Imam University, Kahramanmaras, Turkey; b Endocrinology and Metabolism Division, Biruni University Faculty of Medicine, İstanbul, Turkey; c Faculty of Medicine, Department of Internal Medicine, Kahramanmaras Sutcu Imam University, Kahramanmaraş, Turkey; d Department of Pulmonology, Kahramanmras Necip Fazil State Hospital, Kahramanmaras, Turkey; e Department of Endocrinology and Metabolism Liv Hospital, Gaziantep, Turkey.

**Keywords:** gestational diabetes, inflammation, neutrophil–lymphocyte ratio

## Abstract

Gestational diabetes mellitus (GDM) is a condition that is very common during pregnancy and has negative consequences for both mother and fetus. Insulin resistance has been shown as an important cause in the pathogenesis of GDM and low-level inflammation is suggested to be one of the underlying causes of insulin resistance. We aimed to investigate whether the neutrophil–lymphocyte ratio (NLR), which is an indicator of systemic inflammation, is a predictor for GDM. A total of 228 pregnant women, including 128 GDM (patient group) and 100 healthy pregnant were included in the study. GDM was diagnosed with a 1-step approach between 24 and 28 weeks of pregnancy. We found a significant increase in NLR in the 1st and 3rd trimesters in the GDM group compared to healthy pregnant women, which supports that systemic inflammation starts in the early stages of pregnancy and continues throughout pregnancy. We also reported a positive correlation between NLR and fasting plasma glucose and body mass index in both trimesters. We showed that first trimester NLR independently predicted the development of GDM.

**IMPACT STATEMENT**

**What is already known on this subject:** Gestational diabetes mellitus (GDM) develops on the basis of insulin resistance and inflammation is 1 of the factors responsible for the development of insulin resistance. The risk of diabetes in pregnancy often increases after the first trimester, but it is known that some inflammatory markers increase even in the first trimester in high-risk patients.

**What do the results of this study add?**: We found a significant increase in neutrophil–lymphocyte ratio (NLR) in the 1st and 3rd trimesters in the GDM patients and first trimester NLR independently predicted the development of GDM.

**What are the implications of these findings for clinical practice and/or further research?**: NLR, which is a simple, easy, and inexpensive parameter, can be used in the identification and early diagnosis of pregnant women with high risk of GDM

## 1. Introduction

Gestational diabetes mellitus (GDM) is a disease that occurs during pregnancy due to changes in pregnancy and its frequency is increasing in the world. Its prevalence varies between 1% and 14% in studies.^[1]^

GDM often occurs as a result of a beta cell dysfunction that develops on the basis of chronic insulin resistance. Insulin resistance has been shown as an important cause in the pathogenesis of GDM, type 2 diabetes mellitus (DM) and obesity, and low-level inflammation is suggested to be 1 of the underlying causes of insulin resistance. It has been shown that pro-inflammatory cytokines both cause disruption in insulin signaling pathways and reduce insulin secretion from beta cells.^[2]^

Inflammation is 1 of the main components of natural immunity in humans. Inflammation is a process that occurs as a result of cellular damage and includes increased blood flow, capillary dilation, leukocyte infiltration, and the production of some chemical mediators. Although inflammation has a purpose of protecting the body from harmful factors, in some cases it can also cause harm. Chronic low-level inflammation is a pathological condition that occurs in certain conditions such as metabolic syndrome, DM, and cardiovascular diseases, and it is important in the pathogenesis and progression of diseases.^[3]^ It is known that some markers such as high-sensitivity C-reactive protein (hsCRP), TNF-alpha, interleukin-6, and procalcitonin increase in the presence of low-level inflammation. The systematic inflammatory response is accompanied by changes in circulating white blood cells, and in this case, a relative lymphocytopenia is often observed with neutrophilia.^[4]^ Therefore, in addition to the inflammation markers mentioned above, it has been reported that the neutrophil–lymphocyte ratio (NLR) is an indicator of inflammation and may increase in the presence of subclinical inflammation.

It is important to identify GDM patients as they have important consequences for both the pregnant woman and the fetus. Especially the detection of high-risk pregnants and their proper follow-up reduce morbidity in mother and fetus. Therefore, we aimed to investigate whether the NLR, which is a simple, easily calculated, and inexpensive index of systemic inflammation, is a predictor for gestational diabetes in pregnant women.

## 2. Material and methods

This study is a case–control study conducted in a University Hospital. The study was approved by the decision of KSU Ethics Committee dated 08.06.2021 and numbered 03 and written consent forms were obtained from all participants.

### 2.1. Study design and inclusion criteria

The study included 128 pregnant women who applied to KSU Faculty of Medicine Endocrinology outpatient clinic between January 2018 and June 2021. The patients were diagnosed with GDM at 24–28 weeks. The data of patients that diagnosed with GDM were scanned retrospectively, and the patients with first trimester data were included in the study. In addition, 100 healthy pregnant women, who were in the same age range and had 1st and 3rd trimester data, were included in the study as the control group.

GDM was diagnosed with a 1-step approach between 24 and 28 weeks of pregnancy. G power was used for sample size.^[5]^ Demographic characteristics of pregnant women included in the study; parity, macrosomic infant history, and family history were recorded. In addition, anthropometric (height, weight, body mass index [BMI]) and laboratory data (fasting plasma glucose [FPG], lipid profile, hematological parameters) of all participants were recorded.

### 2.2. Exclusion criteria

Patients with prepregnancy diabetes and those who used cigarettes and alcohol were excluded from the study. Other exclusion criteria included liver failure, kidney failure, hypothyroidism, hyperthyroidism, diseases that would severely limit life expectancy (terminal cancer), drug use that would affect glucose metabolism, morbid obesity, mental retardation, and serious psychiatric illness. Women under the age of 18 and over the age of 45 were not included in the study. The participants without first trimester data were excluded from the study.

### 2.3. Anthropometric measurements

Body mass index was measured with light clothing to at least 0.1 kg accuracy. Height (m) was measured barefoot and standing to at least 0.1 cm accuracy using a wall-mounted gauge. Body mass index was measured as weight/height^2^ (kg/m^2^).

### 2.4. Biochemical measurements

Blood samples were taken between 08:00 am and 09:00 am after 8–10 hours of fasting. Glucose and lipid profile were measured with the spectrophotometric method using the Advia 1800 Chemistry System (Siemens, Germany) and the complete blood count was measured with the CellDyn 3700 SL analyzer (Abbott Diagnostics, IL) in the biochemistry laboratory of the KSU Faculty of Medicine.

Normal reference values were as follow; total cholesterol (T-C) 0–200 mg/dL, triglyceride (TG) 0–150 mg/dL, high-density lipoprotein (HDL) 26–86 mg/dL, low-density lipoprotein (LDL) 0–130 mg/dL, leukocytes 4–10 K/µL, neutrophils 2.00–7.00 K/µL, lymphocytes 0.80–4.00 K/µL, monocytes 0.12–1.20 K/µL, platelet count 150–400 K/µL, and PDW 9.5–15.5 fL, and MPV 6.5–12 fL.

#### 2.5. Gestational diabetes diagnosis

GDM was diagnosed with a 1-step approach at 24–28 weeks of pregnancy. In the 1-step approach; glucose levels were measured at fasting, 1st and 2nd hours after at least 8–10 hours of fasting, following 75 g of glucose mixed in 300 mL of water. High detection of at least 1 of these measured values (FPG ≥92 mg/dL, OGTT 1st hour PG ≥180 mg/dL, and OGTT 2nd hour PG ≥153 mg/dL) was accepted as a diagnosis of GDM.

#### 2.6. Statistical analysis

The study data were evaluated with Shapiro–Wilk test whether the study data were normally distributed. In normally distributed data, independent 2 sample *t* test was used for comparing 2 groups. In non-normally distributed data, Mann–Whitney *U* test, a nonparametric test, was used to compare 2 groups. Chi-square test was used to evaluate the relationship between the frequency distributions of categorical variables. Direct relationship between variations was evaluated with Spearman and Pearson correlation test. Logistic regression analysis was used to determine the effects of variables on GDM. Relative odds were expressed as odds ratio (OR) and confidence interval (CI). The values of the predicted NLR, BMI, and FPG levels and 95% CIs were computed. Descriptive statistics were expressed as mean + standard deviation. Statistical significance was accepted as *P* < .05. Data were analyzed using IBM Statistical Package for Social Sciences (SPSS, Armonk, NY, USA) version 25 package program.

## 3. Results

### 3.1. Sociodemographic data of the patient and control groups

The demographic characteristics of the 228 pregnant (GDM and healthy) included in the study are shown in Table 1. The average age of the GDM group was 32.41 ± 5.73 and the control group was 31.03 ± 5.39 (*P* = .063). The rate of macrosomic birth, family history of DM, and multiparity was also significantly higher in the GDM group (*P* < .001).

**Table 1:** Comparison of demographic data and 1st trimester BMI and laboratory data of pregnant women with gestational diabetes and healthy control group.

Parameters	GDM group (n = 128)	Control group (n = 100)	*P*
Age (year)*	32.41 ± 5.73	31.03 ± 5.39	.063
BMI (kg/m^2^)*	28.30 ± 3.56	25.18 ± 2.87	**<.001**
<25 kg/m^2^ (n, %)†	19 (28.8)	47 (71.2)	**<.001**
25–30 kg/m^2^ (n, %)†	69 (59)	48 (41)	
≥30 kg/m^2^ (n, %)†	40 (88.9)	5 (11.1)	
Family history of DM, n (%)†	59 (80.8)	14 (19.2)	**<.001**
History of macrosomic birth, n (%)†	19 (90.5)	2 (9.5)	**<.001**
Multiparity, n (%)†	35 (70)	15 (30)	**<.001**
FPG (mg/dL)‡	94.97 ± 11.47	82.47 ± 6.34	**<.001**
T-C (mg/dL)‡	205.17 ± 38.53	175.99 ± 24.40	**<.001**
LDL (mg/dL)‡	118.34 ± 44.73	106.86 ± 28.48	**.019**
HDL (mg/dL)‡	48.12 ± 8.20	49.39 ± 9.25	.279
TG (mg/dL)‡	206.55 ± 64.85	109.26 ± 43.63	**<.001**
WBC (/µL)‡	8.44 ± 1.42	7.40 ± 1.64	**<.001**
Neutrophil (/µL)‡	5.50 ± 1.39	4.41 ± 1.43	**<.001**
Lymphocyte (/µL)‡	2.19 ± 0.55	1.97 ± 0.51	**.002**
NLR‡	2.80 ± 1.12	2.24 ± 0.80	**<.001**
Monocytes (/µL)‡	0.55 ± 0.19	0.51 ± 0.16	**.047**
Platelets (×1000/mm^3^)‡	271.47 ± 66.03	285.80 ± 67.03	.108
PLR‡	128.90 ± 37.48	153.02 ± 49.82	**<.001**
PDW (%)‡	14.48 ± 4.06	13.23 ± 3.96	.319
MPV (fL)‡	10.27 ± 1.27	10.00 ± 1.01	.084

Continuous variables were expressed as the mean ± SD; categorical variables were expressed as a number (percentage).

BMI = body mass index, DM = diabetes mellitus, FPG = fasting plasma glucose, GDM = gestational diabetes mellitus, HDL = high-density lipoprotein, LDL = low-density lipoprotein, MPV = mean platelet volume, NLR = neutrophil to lymphocyte ratio, PDW = platelet distribution width, PLR = platelet to lymphocyte ratio, T-C = total cholesterol, TG = triglyceride, WBC = white blood cells.

* Independent samples *t* tests.

† Chi-square *χ*^2^ test.

‡ Mann–Whitney *U* test.

### 3.2. Evaluation of 1st and 3rd trimester metabolic data of the patient group and control group

BMI was significantly higher in the GDM group both in the 1st trimester and in the 3rd trimester (28.30 ± 3.56/25.18 ± 2.87 vs 30.23 ± 3.56 vs 27.11 ± 2.94 respectively, *P* < .001). In addition, the rate of being overweight/obese was significantly higher in the GDM group in both trimesters (*P* < .001). FPG, TC, and TG were found to be significantly higher in the GDM group both in the 1st trimester and in the 3rd trimester (*P* < .001, *P* < .001, *P* < .001 respectively), and LDL in the 1st trimester (*P* = .019) (Tables 1 and 2). There was a positive correlation between 1st trimester FPG with BMI, T-C, and TG levels (*R* = 0.364, *P* < .001, *R* = 0.204, *P* = .002 and *R* = 0.277, *P* < .001, respectively). In addition, there was a positive correlation between 3rd trimester FPG with BMI and TG levels (*R* = 0.372, *P* < .001 vs *R* = 0.186, *P* = .005).

**Table 2:** Comparison of 3rd trimester BMI and laboratory data of pregnant women with gestational diabetes and healthy control group.

Parameters	GDM group (n = 128)	Control group (n = 100)	*P*
BMI (kg/m^2^)*	30.23 ± 3.56	27.11 ± 2.94	**<.001**
<25 kg/m^2^ (n, %)†	6 (25)	18 (75)	**<.001**
25–30 kg/m^2^ (n, %)†	59 (46.8)	67 (53.2)	
≥30 kg/m^2^ (n, %)†	63 (80.8)	15 (19.2)	
FPG (mg/dL)‡	94.47 ± 14.27	83.57 ± 4.69	**<.001**
T-C (mg/dL)‡	223.99 ± 40.49	207.53 ± 29.62	**.001**
LDL (mg/dL)‡	137.36 ± 41.54	136.84 ± 28.49	.912
HDL (mg/dL)‡	43.30 ± 7.38	44.45 ± 8.32	.279
TG (mg/dL)‡	216.60 ± 103.95	131.11 ± 52.35	**<.001**
WBC (/µL)‡	9.37 ± 1.85	8.09 ± 1.43	**<.001**
Neutrophil (/µL)‡	6.69 ± 1.82	5.40 ± 1.39	**<.001**
Lymphocyte (/µL)‡	2.03 ± 0.50	1.89 ± 0.58	**.043**
NLR‡	3.47 ± 1.17	3.12 ± 1.27	**.036**
Monocytes (/µL)‡	0.63 ± 0.23	0.58 ± 0.16	**.054**
Platelet (×1000/mm^3^)‡	247.59 ± 62.02	251.19 ± 61.61	.663
PLR‡	134.20 ± 65.72	140.23 ± 38.73	.417
PDW (%)‡	14.13 ± 3.06	12.56 ± 2.65	.301
MPV (fL)‡	10.39 ± 1.09	10.34 ± 0.99	.749

Continuous variables were expressed as the mean ± SD; categorical variables were expressed as a number (percentage).

BMI = body mass index, FPG = fasting plasma glucose, GDM = gestational diabetes mellitus, HDL = high-density lipoprotein, LDL = low-density lipoprotein, MPV = mean platelet volume, NLR = neutrophil to lymphocyte ratio, PDW = platelet distribution width, PLR = platelet to lymphocyte ratio, T-C = total cholesterol, TG = triglyceride, WBC = white blood cells.

* Independent samples *t* tests.

† Chi-square *χ*^2^ test.

‡ Mann–Whitney *U* test.

### 3.3. Comparison of the 1st and 3rd trimester hematological data of the patient group and control group

**Figure 1.**
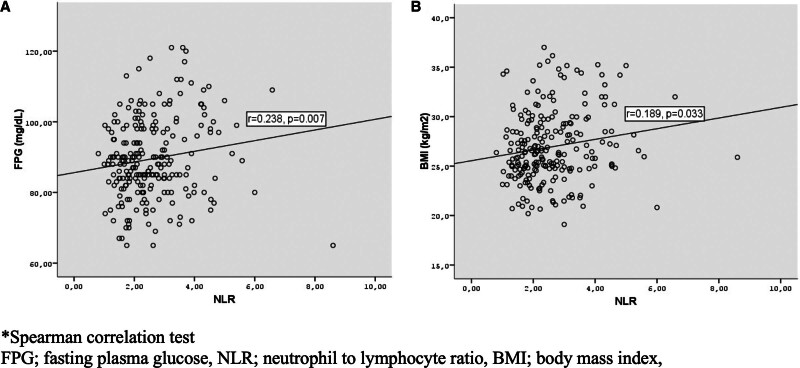
The relationship between neutrophil lymphocyte ratio and FPG (A) and BMI in the first trimester. *Spearman correlation test. BMI = body mass index, FPG = fasting plasma glucose, NLR = neutrophil to lymphocyte ratio.

**Figure 2.**
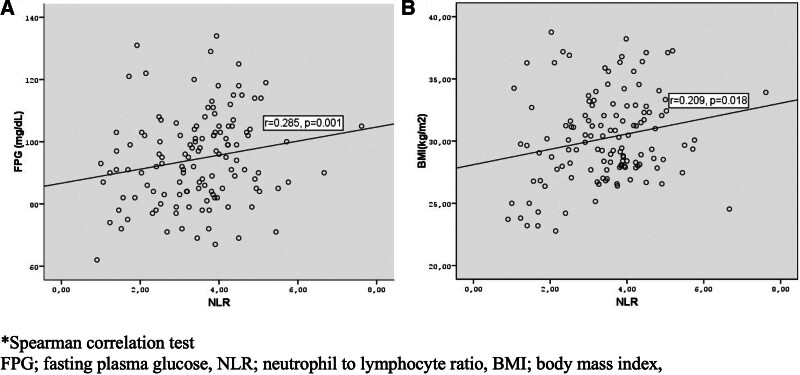
The relationship between neutrophil lymphocyte ratio and FPG (A) and BMI in the third trimester. *Spearman correlation test. BMI = body mass index, FPG = fasting plasma glucose, NLR = neutrophil to lymphocyte ratio.

When GDM and control groups were compared in terms of hematological parameters (Tables 1 and 2)WBC, neutrophil, lymphocyte, monocyte count, and NLR were higher significantly in the GDM group both in the 1st and 3rd trimester (*P* < .001, *P* < .001, *P* = .002, *P* = .047, *P* < .001 and *P* < .001, *P* < .001, *P* = .043, *P* = .054, *P* = .036, respectively). There was a positive correlation between both 1st trimester and 3rd trimester NLR with FPG levels and BMI (*R* = 0.238, *P* = .007, *R* = 0.189, *P* = .033 and *R* = 0.285, *P* = .001, *R* = 0.209, *P* = .018, respectively) (Figs. 1 and 2).

### 3.4. Logistic regression and receiver operating characteristic analysis

In the multiple logistic regression model using an enter method, 1st trimester NLR (OR = 1.512, *P* = .049), FPG (OR = 1.125, *P* < .001), and 3st trimester BMI (OR = 1.215, *P* = .017), FPG (OR = 1.050, *P* = .019) still remained significant predictors of GDM after adjusting for the confounding variables (Table 3).

**Table 3:** Factors affecting the development of gestational diabetes.

Risk factors	OR	95% CI	*P*
NLR (1st trimester)	1.512	1.003–2.280	**.049**
FPG (1st trimester)	1.125	1.072–1.180	**<.001**
BMI (3rd trimester)	1.215	1.035–1.425	**.017**
FPG (3rd trimester)	1.050	1.008–1.093	**.019**

All the variables (family history of DM, macrosomic birth, multiparity, BMI, FPG, T-C, LDL, TG, NLR, PLR) related to gestational diabetes mellitus were examined and only those significant at *P* < .05 level are used in multiple logistic regression analysis.

Nonsignificant variables were not indicated in the table.

BMI = body mass index, CI = confidence interval, DM = diabetes mellitus, FPG = fasting plasma glucose, LDL = low-density lipoprotein, NLR = neutrophil to lymphocyte ratio, OR = odd ratio, PLR = platelet to lymphocyte ratio, T-C = total cholesterol, TG = triglyceride.

**Figure 3.**
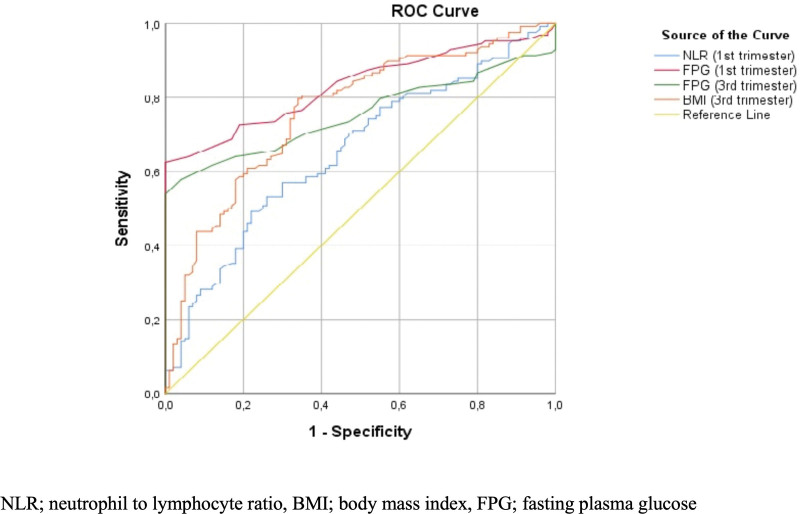
Receiver operator characteristic (ROC) curve of risk factors to predict GDM. BMI = body mass index, FPG = fasting plasma glucose, GDM = gestational diabetes mellitus, NLR = neutrophil to lymphocyte ratio.

This receiver operating characteristic curve shows risk factors to predict GDM (Fig. 3). The optimal cutoff point of 1st trimester NLR in the prediction of GDM was 2.20/µL, with a specificity of 66.4% and sensitivity of 55.0% (AUC = 0.653, *P* < .001).

The cutoff for 1st trimester FPG is 87.5mg/dL (AUC = 0.831, sensitivity 73.4%, specificity 72%, *P* < .001), and the cutoff for 3st trimester FPG is 85.5 mg/dL (AUC = 0.754, sensitivity 70.3%, specificity 64%, *P* < .001) was detected. In addition, the cutoff for 3rd trimester BMI (between 24–28 weeks) was 27.7 kg/m^2^ (AUC = 0.761, sensitivity 77.3%, specificity 67%, *P* < .001) (Table 4).

**Table 4:** ROC analysis of factors affecting the development of gestational diabetes.

Risk factors	AUC (95%)	Cutoff	*P*	Sensitivity	Specificity
NLR (1st trimester)	0.653 (0.582–0.724)	2.20	**<.001**	66.4	55.0
FPG (1st trimester)	0.831 (0.777–0.884)	87.5	**<.001**	73.4	72.0
FPG (3rd trimester)	0.754 (0.690–0.819)	85.5	**<.001**	70.3	64.0
BMI (3rd trimester)	0.761 (0.699–0.824)	27.7	**<.001**	77.3	67.0

AUC = area under curve, BMI = body mass index, FPG = fasting plasma glucose, NLR = neutrophil to lymphocyte ratio.

## 4. Discussion

GDM is a condition that negatively affects the health of both the fetus and the mother during pregnancy. Being obese or overweight is 1 of the important risk factors for GDM, which leads to an increase in metabolic risk factors and an increase in the frequency of GDM in patients. In a study, GDM was detected at a rate of 20.4% in patients with high first trimester Body Mass Index (BMI), while it was observed at a rate of 12.6% in patients with a normal BMI.^[6]^ In another study, more GDM was found in patients with high BMI and insulin therapy was more needed in these patients because of GDM.^[7]^ Similar to the literature, we showed that both being overweight/obese before pregnancy and increased BMI during pregnancy are important risk factors for the development of GDM. In our study, we found that the BMI of GDM patients was significantly higher than healthy pregnant women both in the first trimester (28.30 ± 3.56 vs 25.18 ± 2.87) and in the third trimester (30.23 ± 3.56 vs 27.11 ± 2.94). When we compared the patient and control groups according to BMI, we found GDM in 28.8% of the participants with a normal BMI and in 88.9% of those with a BMI above 30 kg/m2. We also found that there was a significant positive correlation between FPG and BMI. We showed that a BMI above 27.7, especially between 24 and 28 weeks, is an independent risk factor for the development of GDM.

In our study, when we compared the GDM group with the healthy group, we found that family history of DM, multiparity, previous macrosomia, and GDM history were significantly higher in the GDM group. In the literature, it is reported that the history of macrosomia, history of GDM, and congenital anomaly in previous pregnancies significantly increase the risk of GDM. In a study, it was shown that the risk of GDM increased 8 times in those with a previous history of GDM and 4 times in those with macrosomia. At the same time, similar to our findings, studies have shown that multiparity and family history of DM increase the risk of GDM.^[8]^

It has been shown that some metabolic parameters change in patients with GDM even in the early stages of pregnancy. In a study, it was reported that patients diagnosed with GDM at 24–28 weeks had higher TG, LDL-C and T-C levels, and lower HDL-C levels, even in the early stages of the 2nd trimester, compared to patients who did not develop GDM.^[9]^ In another study, TG and TG/HDL cholesterol ratios were found to be higher and HDL cholesterol levels to be lower in GDM patients during the whole pregnancy.^[10]^ In our study, T-C and TG levels were found to be significantly higher in GDM patients compared to healthy pregnant women both in the 1st trimester and in the 3rd trimester, and LDL were significantly higher in the 1st trimester. There was also a significant positive correlation between FPG and T-C and TG levels. Our findings support that the lipid profile is negatively affected both in the early and late stages of GDM.

As there are changes in many systems during pregnancy, changes occur in the immune system as well. A low-grade systemic inflammation occurs during pregnancy. While the innate immune system is more activated in normal pregnancy, the adaptive immune system that occurs later is suppressed in order to prevent fetal rejection. During pregnancy, the T helper (Th) cell profile shifts from the pro-inflammatory Th1 profile to the anti-inflammatory Th2 profile, and this change is important for fetal protection.^[11]^ There is conflicting information in the literature regarding the changes in hematological parameters in pregnant women with GDM. In their recent study, Hassan et al^[12]^ found no significant difference in hematological parameters in patients who were followed up from the early period to the end of pregnancy, with and without GDM. However, Yilmaz et al^[13]^ reported that white blood cell, platelet, neutrophil, lymphocyte count, mean platelet volume, and red cell distribution width were significantly higher in GDM patients compared to the control group. They also showed that first trimester mean platelet volume and red cell distribution ratio were associated with the development of GDM. Fagninou et al^[14]^ found that total leukocyte, lymphocyte, and platelet counts were significantly higher in GDM patients. In our study, we found significantly higher WBC, neutrophil, lymphocyte, and monocyte counts in pregnant women with GDM compared to healthy women in both the 1st and 3rd trimesters. When our findings are evaluated together with the literature, it suggests that hematological parameters, which may be indicative of systemic inflammation, should be carefully evaluated throughout pregnancy.

Insulin resistance, which occurs in the 2nd and 3rd trimester, is often blamed for the pathogenesis of GDM. In some studies, it has been shown that insulin resistance decreases towards the end of the first trimester, but gradually increases thereafter.^[15]^ Therefore, screening for GDM is performed in the second or third trimesters. However, it has been suggested that some parameters that can predict GDM in the first trimester can be used in patients. In a study, it has been shown that CRP levels in the first trimester are found to be significantly higher in those who will develop GDM in the future.^[16]^ Kansu-Celik et al^[17]^ showed that high hs-CRP, high fasting blood sugar, and low fetuin-A levels in the first trimester predict future GDM early. In another study, it was reported that the risk of GDM is higher in pregnant women with high neutrophil values in the first trimester.^[18]^ But another study found that first trimester NLR values were insignificant between GDM and normal patients, Platelet/lymphocyte rate was the only significant value at first trimester.^[19]^ We found a significant increase in NLR in the 1st and 3rd trimesters in the GDM group compared to healthy pregnant women, which supports that systemic inflammation starts in the early stages of pregnancy and continues throughout pregnancy. We also reported a positive correlation between NLR and FPG and BMI in both trimesters. We showed that first trimester NLR independently predicted the development of GDM even when other confounding factors were excluded and a cutoff ratio of 2.2 (66.4% sensitivity and 55% specificity) could be used.

There were some limitations in the study. The data of the patients were obtained during the OGTT screening between 24 and 28 weeks and those with first trimester data were included in the study, therefore the 2nd trimester data of the patients could not be evaluated. Other inflammation markers such as interleukin-6 and hsCRP were not evaluated and a possible relationship with these markers and NLR was not investigated. Another limitation of the study was the relatively low sensitivity and specificity of NLR, some conditions such as the presence of other inflammatory diseases may limit its usage further.

In conclusion, GDM is a metabolic disease with adverse consequences for the mother and fetus. Systemic inflammation plays an important role in the development of GDM. In this study, we showed that NLR, which is a simple, easy, and inexpensive parameter, can be used in the identification and early diagnosis of pregnant women with high risk of GDM. In addition, we think that weight gain may create an additional risk for the development of GDM by increasing systemic inflammation, and lifestyle changes for weight control should be planned from the first trimester of pregnancy.

## Author contributions

**Conceptualization:** Murat Sahin, Dilek Tüzün, Songül Işiktaş, Hatice Şahin.

Data curation: Murat Sahin.

**Formal analysis:** Murat Sahin, Ayten Oğuz, Dilek Tüzün, Cansu Ülgen.

**Investigation:** Murat Sahin, Okay Işiktaş, Cansu Ülgen, Kamile Gül.

**Methodology:** Murat Sahin, Dilek Tüzün, Okay Işiktaş, Songül Işiktaş, Hatice Şahin.

**Project administration:** Murat Sahin.

**Resources:** Murat Sahin.

**Software:** Murat Sahin.

**Visualization:** Ayten Oğuz, Kamile Gül.

**Writing – original draft:** Murat Sahin, Okay Işiktaş, Songül Işiktaş, Cansu Ülgen, Hatice Şahin, Kamile Gül.

**Writing – review & editing:** Murat Sahin, Ayten Oğuz, Dilek Tüzün, Cansu Ülgen, Kamile Gül.
